# Gender is not simply a matter of black and white, or is it?

**DOI:** 10.1098/rstb.2017.0126

**Published:** 2018-06-18

**Authors:** Gün R. Semin, Tomás Palma, Cengiz Acartürk, Aleksandra Dziuba

**Affiliations:** 1 William James Center for Research, ISPA - Instituto Universitário, Rua Jardim do Tabaco 41, 1149-041 Lisboa, Portugal; 2 Department of Psychology, Utrecht University, 3584 CS Utrecht, The Netherlands; 3 CICPSI, Faculdade de Psicologia, Universidade de Lisboa, 1649-013 Lisboa, Portugal; 4 Informatics Insitute, Department of Cognitive Sciences, Middle East Technical University, 06800 Ankara, Turkey

**Keywords:** grounding gender, colour and gender categorization, eye tracking, disambiguating amorphous stimuli

## Abstract

Based on research in physical anthropology, we argue that brightness marks the abstract category of gender, with light colours marking the female gender and dark colours marking the male gender. In a set of three experiments, we examine this hypothesis, first in a speeded gender classification experiment with male and female names presented in black and white. As expected, male names in black and female names in white are classified faster than the reverse gender-colour combinations. The second experiment relies on a gender classification task involving the disambiguation of very briefly appearing non-descript stimuli in the form of black and white ‘blobs’. The former are classified predominantly as male and the latter as female names. Finally, the processes driving light and dark object choices for males and females are examined by tracking the number of fixations and their duration in an eye-tracking experiment. The results reveal that when choosing for a male target, participants look longer and make more fixations on dark objects, and the same for light objects when choosing for a female target. The implications of these findings, which repeatedly reveal the same data patterns across experiments with Dutch, Portuguese and Turkish samples for the abstract category of gender, are discussed. The discussion attempts to enlarge the subject beyond mainstream models of embodied grounding.

This article is part of the theme issue ‘Varieties of abstract concepts: development, use and representation in the brain’.

Apart from any cultural or nurture-related factors, maybe we're just hard-wired to associate female with ‘fair’. I have a small white fuzzy dog; everybody calls him a ‘she’ even though he rather obviously has a penis. However, no one assumes my small black dog is a ‘she’, ever.

https://www.quora.com/Why-are-women-called-the-fairer-sex

## 1. Introduction

Occasionally, one can observe and infer, or even demonstrate how an abstract category is represented without having to design a carefully crafted experiment to reveal whether an abstract category is made accessible by means of a metaphor or not. A defining feature of the category may simply be there for the cognitive ‘grabbing’. Gender is one such category. You can see how it is grounded when people spontaneously categorize your dog, as in the quote above. You are also likely to observe an uninstructed waiter serving a cappuccino to a female customer and an espresso to a male. There is an assumption that white or bright is associated with female and black or dark with the male. This sensory dimension—bright to dark—is a distinctive feature of gender and gender-related actions [1]. There is historical and anthropological evidence that the gender categories, female–male and the sensory dimension—bright to dark—are associated (e.g. [2–4]). Indeed, sexual dimorphism of skin colour, namely that females have a lighter skin colour than males, is well established in research outside of the psychological literature (e.g. [5–8]). Nevertheless, the link between the physical evidence (sexual dimorphism of skin colour) and the gender marking remains a speculative one.

Below, we start with providing first the background against which the studies we report were conceptualized. This is followed by an overview of the three studies on the pervasive presence of the association between the sensory dimension of brightness–darkness and our representations of gender.

## 2. Background

One of the early contributions to research on the role of Conceptual Metaphor Theory ([9,10]; also see [11]) identified the sensory opposition between brightness and darkness as grounding the abstract category of valence [12]. This led to several studies examining the interface between affect and its spatial representation (e.g. [13–15]). The theoretical anchoring of this work relies on the general proposition that it's a ‘natural’ tendency to ground an abstract category such as valence or affect that cannot be touched, seen or smelled in some sensorimotor experience. This link, then, structures human thought, and shapes the way our cognition deals with it—namely, how we encode, store and retrieve related information. The sensorimotor experience that grounds the category serves as a scaffold for our thinking about abstract concepts such as time (e.g. [16]), justice (e.g. [17]) or morality (e.g. [18,19]).

For instance, Meier *et al*. [20] showed that when participants had to match the brightness of a word to a set of squares varying in shading there is a metaphor-consistent bias in their brightness judgements. Earlier, Meier *et al*. [12] showed that negative words were evaluated faster and more accurately when presented in a black font rather than a white font, and positive words were evaluated faster and more accurately when presented in a white rather than a black font. The assumption underlying these findings is that affective evaluations activate the sensory dimension bright–dark. Accordingly, the representations of abstract categories such as valence or affect are inherently body-based (e.g. [11,21–29]). Metaphors, it is argued, facilitate understanding abstract (target) concepts by resorting to more concrete (source) concepts—and in the domain referred to above, the representation of valence is anchored by the sensory dimension of brightness–darkness.

## 3. Shades of the flesh: sexual dimorphism of skin colour

As is the case with space grounding diverse abstract concepts, the brightness–darkness dimension can be seen in relation to concepts aside from valence, namely gender. This dimension constitutes one in which the representation of males and females differ, but the nature of this relationship is different from the case of valence. Whereas the valence–brightness interface is a metaphoric one, the one between gender and this dimension is not. On the contrary, the brightness dimension is one on which men and women do differ in terms of their skin colour. The difference between the valence differentiation and gender differentiation on the light–dark dimension is that the former is a metaphorical differentiation, whereas the latter is probably derived from the universal dimorphism in skin colour between males and females [5–7]. Sexual dimorphism of skin colour was noted early on in history and goes back not only to early Greeks and Etruscans, but also to the Aztecs, Egyptians, Chinese and Japanese [3,4].

There is a distinctive and universal adaptive pattern to the distribution of skin colour. Jablonski & Chaplin [6] summarize this as follows: ‘Throughout the world, human skin colour has evolved to be dark enough to prevent sunlight from destroying the nutrient folate but light enough to foster the production of vitamin D’ (p. 74). This is underlined by the well-known geographical variation of skin pigmentation and the strong correlation between skin reflectance and latitude, a correlation that is even higher with UVR [6,8,30,31].

However, and more relevant to the argument here: there is a systematic difference in the shades of skin colour between females and males that is orthogonal to geographical variations in skin colour. Let us illustrate: if we were to provide two photos of skin samples, one being of a light shade and the other dark and then ask which one was a female sample and which one was a male sample, then most people are likely to associate the paler one with a woman's skin and the darker one with a male's. Indeed, Jablonski and Chaplin's (e.g. [6]) research findings underline this observation as well as earlier reports of sexual dimorphism in human skin pigmentation (e.g. [2,3]) showing that females are consistently lighter than males in all populations studied ([5,6], p. 600,601). There were diverse reasons advanced for this systematic difference in the sexual dimorphism in skin pigmentation, such as the dimorphism being due to infantile mimicry, sexual selection or a combination of both factors [2,3,32,33]. For instance, Frost [3] suggests that being attracted to young human infants or females is due to their lighter pigmentation and that lighter coloured females are perceived as more attractive and are preferred partners. However, the prevalent view is that sexual dimorphism in skin pigmentation is primarily due to natural selection, on the basis of the need of females to maximize cutaneous vitamin D3 production so that it is possible for them to maintain the higher demands they have for calcium requirements during pregnancy and lactation. In the case of males, it is argued that darker pigmentation has been the object of natural selection because it enables the maintenance of levels of folate that are required to protect sperm production. The sperm production process depends on folate for DNA synthesis. Thus, the disparity in skin colour is due to natural selection. This disparity may be further strengthened by the culturally anchored preference for females that are paler in some societies, such as current day Japan or China. However, one can argue that this preference is a social–cultural translation of the evolved differences in skin colour by males and females and a consequence of the exaggeration of categorical differences [34] that ensues upon making an explicit categorical division [5,6].

## 4. Overview

The question we addressed in the first two of the three experiments reported here was does the marking of gender with the bright–dark dimension influence the classification of male and female names? Namely, does the presentation of male names in a darker typeface compared with a lighter typeface and the presentation of female names in lighter typeface compared with a darker typeface speed up the name classification process? To this end, we, first of all, replicated the first experiment reported by Semin & Palma [1]. This experiment involved a simple classification task in which participants had to decide and indicate, as fast and as accurately as possible, whether a set of male and female names presented in darker or lighter typeface were male or female names. The current study was a replication of the study originally conducted with Dutch participants, but now with Portuguese participants and some minor procedural variations. This permitted us not only to check the robustness of the earlier findings, but also their generalizability to a population from a different culture.

**Figure 1.**
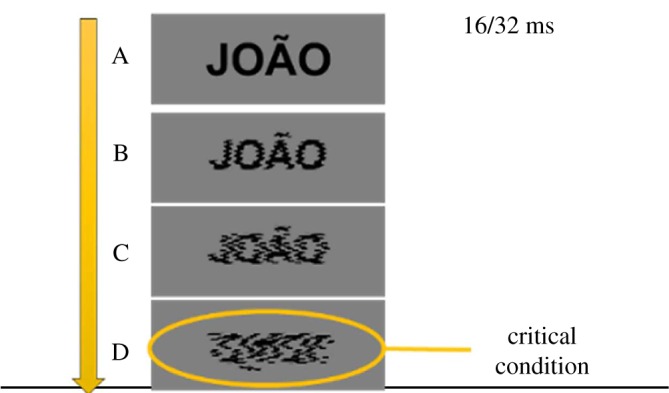
Stimulus presentation in Experiment 2.

In the second experiment, participants had a 16 or 32 ms exposure to four types of male or female ‘name’ stimuli. The stimulus characteristics were controlled for their clarity, ranging from a clear typeface representation (as in figure 1) to the fourth type of representation, which consisted of a distortion representing a mere black or a white ‘blob’ (D in figure 1). The prediction was: if gender is marked by the brightness dimension, then in the case of the ‘blob’ condition participants would more frequently interpret and classify the black blob as a male name and the white blob as a female name.

In the third and final experiment, we adapted an earlier paradigm (see [1], Experiment 4), which involved a preference experiment in which either a male or a female participant had to indicate the object they thought would be chosen by a male or a female target person. The same objects were in either black or white or alternatively dark- or light-colours. The dependent variable in this eye-tracking experiment was gaze duration, number of fixations and object choice. The prediction was that participants would choose the light or white objects for female target persons and vice versa for male target persons. Moreover, it was expected that gaze duration would be longer, and the number of fixations would be higher, for the objects that were chosen because gaze location and visual attention are usually coupled in natural and unconstrained settings [35]. These predictions underlie the argument that the implicitly relevant objects would be more salient and thus attract more attention.

Across *all* the studies reported here, we acknowledge all data exclusions, all manipulations and all measures. The databases and stimuli are available at the Open Science Framework website (https://osf.io/mdv72). The studies were approved by the Institutional Review Boards of the respective universities. All participants provided written informed consent prior to participation and were free to stop at any point in time during the experiment.

## 5. Experiment 1

This study was a slightly modified experiment of that reported by [1], Exp. 1. The aim was to examine the replicability and robustness of the original study that examined the time it took participants to classify male and female names that were presented in black or white against a grey background. The main difference was the cultural background of the participants. While the original study was run with Dutch participants, the current study was run with Portuguese participants. The robustness of the hypothesis under consideration was that male names presented in black would be classified faster than female names presented in black. By contrast, female names presented in white were predicted to be processed faster than those in black.

### (a) Method

#### (i) Participants and stimulus material

Thirty-seven students (27 women, mean age = 24.30; one participant did not report her age) participated as paid volunteers. The experimental stimuli consisted of a set of 24 names, half of which were typical Portuguese male names and the other half female names.

#### (ii) Procedure

The participants took part in a computer-administered task, where they were instructed to classify, as fast and as accurately as possible, whether the name they saw on the monitor was a male or a female name. The experimental session was preceded by 16 practice trials with 4 male names and 4 female names that did not appear in the main experiment. The names were either in black (RGB code: 0, 0, 0) or white (RGB code: 255, 255, 255) letters presented in a random order varying across participants against a grey background (RGB code: 128, 128, 128).

In the practice trials and the main experiment, participants saw a fixation cross in the middle of the monitor against a grey background. The fixation cross was presented for random durations between 250 to 1000 ms. Then, a name appeared and stayed on the screen until a response was given. Half of the participants used the right key (M) to classify the male names and the left key (X) to classify the female names. The key-labels were reversed for the other half of the participants. After each response, there was a grey screen for 500 ms. Participants were instructed to be as fast and accurate as possible. Participants were thanked and debriefed after the experiment was over.

The experiment consisted of three blocks, each composed of 48 trials (24 names × 2 colours). This was done to examine the ‘time course’ of the responses and the response pattern. The question was whether the predicted interaction would emerge only in the first trial block, or at a later block, or whether it would be independent of the block manipulation.

Because we expected a high degree of accuracy in this study (given the easiness of the task), our main interest was in reaction times. Thus, the dependent variable in this experiment was the mean reaction time required to classify the name on the monitor as male or female.

The differences between this study and original one (Exp. 1, [1]) were: in the Dutch study, we (a) used 20 male and 20 female Dutch names; (b) gave participants a 800 ms response window; (c) had fewer trials. Moreover, we had (d) differences in the lab settings (e.g. response boxes versus keyboard, the lighting of the experimental rooms). Finally, the obvious difference was in the cultural background of the participants.

### (b) Results and discussion

In this and the following experiments, we report partial eta-squares 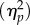 as effect sizes for omnibus ANOVA tests and Cohen's *d_z_ (t/√N*; [36]) for planned *t*-test comparisons on within variables. We set the significance criterion at *p* < 0.050.

Reaction times to the names were analyzed after removing errors (4%) and outliers. Outlier removal was done in two steps. First, reaction times below 200 ms and above 1200 ms were removed (2%). Second, separately for each participant, we calculated the absolute deviations around the median (MAD) for each block [37]. Reaction times below or above 2.5 MADs from the median of the respective block were then excluded (5%). The median absolute deviation (MAD) is considered a very robust method to detect outliers because it is relatively more insensitive to the presence of extreme outliers and sample size than the mean plus or minus some standard deviation method (see [37]). We noticed that one participant had a high percentage of errors (14%) compared with the overall percentage of errors (4%). This percentage increased substantially (35%) after the outlier removal procedures and we therefore excluded this participant's data from all analyses. The analyses reported below were thus conducted on data from 36 participants.

#### (i) Reaction times

The data were analyzed in a 2 (names: male versus female) × 2 (colour: black versus white) × 3 (blocks: block 1 versus block 2 versus block 3) repeated measures ANOVA. The interaction between names and colour was significant, *F*_1,35_ = 8.87, *p* = 0.005, 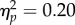. As predicted, male names in black were classified significantly faster (*M* = 524 ms, s.e. = 22 ms) than male names in white (*M* = 532 ms, s.e. = 22 ms), *t*_35_ = 2.53, *p* = 0.016, *d_z_* = 0.42. The reverse was observed for female names, with white female names being classified significantly faster (*M* = 517 ms, s.e. = 23 ms) than black female names (*M* = 525 ms, s.e. = 21 ms), *t*_35_ = 2.15, *p* = 0.039, *d_z_* = 0.36. Results also showed a significant three-way interaction, *F*_1,35_ = 8.87, *p* = 0.005, 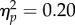. Table 1 shows the data pattern. To interpret this interaction, we tested for the presence of an interaction between names and colour in each of three blocks. Planned contrast analysis revealed no significant interaction between names and colour in blocks 1 and 2 *F*_1,35_ = 1.50, *p* = 0.229, 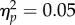; *F*_1,35_ = 0.01, *p* = 0.916, 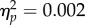. However, a strong interaction between names and colour was observed in the third block, *F*_1, 35_ = 15.54, *p* < 0.001, 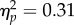, with male names in black being responded to faster than male names in white, *t*_35_ = 2.39, *p* = 0.022, *d_z_* = 0.40, and white female names being responded to faster than black female names, *t*_35_ = 3.42, *p* = 0.002, *d_z_* = 0.57.

**Table 1.** Mean reaction times in milliseconds (and standard errors) as a function of colour, gender of name and block (Experiment 1).

	Block 1		Block 2		Block 3	
	colour		colour		colour	
names	black	white	black	white	black	White
male	529 (15)	538 (17)	525 (14)	525 (13)	518 (13)	532 (13)
female	531 (16)	531 (17)	516 (12)	515 (14)	526 (12)	506 (12)

None of the main effects reached statistical significance. The counterbalancing of the response keys did not moderate the results presented above. In this experiment and in Experiment 2, we did not test for the moderating role of participants' gender because both samples were extremely unbalanced in terms of gender, with many more females than males. In Experiment 3, we had the same number of females and males in our sample.

The first experiment replicated our earlier findings ([1], Exp. 1) with a Portuguese sample, underlining the robustness of the original findings. Additionally, results suggested that our effect unfolds in a somewhat asymmetrical way. Namely, while for male names the effect of colour seems to be present from the first trials onwards, for female names the effect seems to require more trials to build up (table 1). This pattern of results is congruent with our previous work in which, using fewer trials than in the current experiment, we found a stronger effect for male than female names (see [1], Exp. 1).

## 6. Experiment 2

In the second experiment, we further explored this relationship between gender and colour using a different task. Namely, in this experiment our main goal was to test whether participants would use colour to infer the gender of names. For this purpose, we employed what one may term a *disambiguation bias task*. Participants were provided with a set of four different versions of the stimuli in black and white (figure 1). The stimuli included male or female names and nouns that were presented in either black or white against a medial-grey background. The male and female names were presented in black and white under two stimulus exposure conditions (16 and 32 ms). The name stimuli were displayed in four presentation conditions (figure 1), namely: (A) perfectly visible/readable, (B) blurred, (C) very blurred and (D) unreadable (black or white blob). Consequently, we had three different types of representations of each name and a blob in white and black. In the case of condition A, all the name stimuli were perfectly readable. They became progressively more scrambled from B to C, all the way down to the version that was entirely ambiguous. Condition D was not only scrambled but also reversed upside-down so as to avoid any possible readable connection to the original stimuli.

The main dependent variable in this experiment was classification of these blob stimuli into male or female. The prediction was that participants would use colour to infer the gender of blobs. Specifically, black blobs would be classified more frequently as male ‘names’ and white blobs would be classified as female ‘names’. Because we expected a high level of accuracy in the three remaining presentation conditions (i.e. A, B and C), despite the short presentation times, we also analysed participants' reaction times to these names. We expected that the participants would be faster for black male and white female names compared with white male and black female names, thus replicating our previous findings.

### (a) Method

#### (i) Participants and stimulus material

Thirty-six students (29 women, mean age = 20.22) participated in exchange for course credit. The experimental stimuli were 24 typical Portuguese names (12 male names and 12 female names), some of which were used in Experiment 1 and some were new names.

#### (ii) Procedure

The experiment was computer administered. The participants’ task was to classify, as fast and as accurately as possible, whether the name they saw on the monitor was a male or a female name by pressing one of two keys on a button-box (counterbalanced). The experimental session was preceded by a practice set that comprised 20 names in black or white. The set had a randomized combination of two male names, two female names and four nouns. These were not used in the main experiment. During the practice trials, participants received feedback only when they were slow (too slow).

The main part of the experiment started after the practice set and consisted of a total of 576 trials presented in three blocks of 192. The 576 trials resulted from the sets of 12 males and 12 females and 12 nouns (as dummy items). The nouns were selected from Prada & Santos [38] and Garcia-Marques [39], and matched the names in terms of valence, first letter, gender and number of letters.

All sets were presented in black and white and in the four presentation conditions (from A to D) presented in a random order varying across participants against a grey background. Finally, the stimuli were presented for either a duration of 16 or 32 ms on the monitor. It is important to note that the ambiguous blob-like version was manipulated directly from the same source, so even though there was nothing to read, the amount of sensorial information (in this case, ‘blackness’ or ‘whiteness’) was the same. Each response was followed by a grey screen for 500 ms.

The dependent variable was the number of male and female name classifications in the case of the blobs (presentation condition 4) and reaction times in the case of the remaining presentation conditions.

Participants were thanked and debriefed after the experiment was over.

### (b) Results and discussion

#### (i) Disambiguation bias (presentation condition D)

Since male and female responses are mutually dependent, we calculated the average proportion of times that each blob was judged to be a male name, with 50% indicating an equal number of male and female responses, 100% indicating only male responses and 0% indicating no male responses (i.e. 100% of female responses; see [40] and [41] for similar statistical analysis). These proportions were then submitted to a 2 (colour: black versus white) × 2 (time: 16 ms versus 32 ms) repeated measures ANOVA, which revealed only a strong main effect of colour, *F*_1,35_ = 26.61, *p* < 0.001, 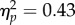. As predicted, black blobs (*M* = 0.54, s.e. = 0.04) were more frequently disambiguated as a male name than were white blobs (*M* = 0.46, s.e. = 0.04). No other effects reached statistical significance (all *F*s < 1). The counterbalancing of the response keys did not moderate this result.

#### (ii) Reaction times (presentation conditions A, B and C)

Before analysing participants' reaction times, we removed trials with response errors (18%) and outliers. Outlier removal was done following the same procedures employed in the previous experiment. Namely, first, we discarded reaction times below 200 ms and above 1200 ms (6%) and then reaction times below and above 2.5 MADs (4%; [37]).

To simplify the interpretation of the results, we performed separate analyses for names and for nouns. Regarding the names, the data were analysed in a 2 (names: male versus female) × 2 (colour: black versus white) × 2 (time: 16 versus 32) × 3 (presentation conditions: A versus B versus C) repeated measures ANOVA. Results revealed that the predicted interaction between names and colour was statistically significant, *F*_1,35_ = 4.96, *p* = 0.032, 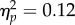. Planned comparisons showed that, indeed, male names in black were classified significantly faster (*M* = 497 ms, s.e. = 35 ms) than male names in white (*M* = 508 ms, s.e. = 35 ms), *t*_35_ = 2.77, *p* = 0.009, *d_z_* = 0.46, but that the difference between white female names (*M* = 497 ms, s.e. = 33 ms) and black female names (*M* = 495 ms, s.e. = 33 ms) did not reach statistical significance (*t* < 1). There were other theoretically uninteresting results, which we do not report here for reasons of space.

Regarding the nouns, a 2 (nouns: male versus female) × 2 (colour: black versus white) × 2 (time: 16 versus 32) × 3 (presentation conditions: A versus B versus C) repeated measures ANOVA showed no interaction between nouns and colour (*F* < 1). The counterbalancing of the response keys did not moderate any of the results presented above (see endnote 1).

The results of the critical condition revealed the predicted *disambiguation bias*: when participants were presented with black and white blobs they were more likely to classify the black blobs as male names and the white blobs as female names. These findings underline further the predicted interface between the brightness dimension and the role it plays in marking gender. The results for the reaction times, however, revealed a weak pattern as obtained in previous experiments, namely, we found the predicted results for male but not for female names. One reason for this weak pattern may be related to the specific features of the disambiguation task and its requirements. Although we instructed participants to respond faster, which they did, this task was much more difficult and, therefore, gave rise to substantially more errors than the classification task used in the previous experiments. Thus, in such a task, the number of errors is a more adequate criterion or measure than reaction times [12]. Future research should, however, put this speculation to test and generalize our findings to other reaction time tasks.

## 7. Experiment 3

Experiment 3 was designed to examine how the brightness dimension shapes process features when people choose between two objects for either a male target person or a female target person. We examined what people looked at, and how long they looked at it, given a choice between two objects when asked to make a choice for either a male or a female target. Is there a distinctive gender-driven preference when confronted with choices that are either dark or light consumer items? The hypothesis we pursued was that participants would manifest longer gaze durations and higher numbers of fixations on the brighter objects compared with the darker objects when choosing an object for a female target and vice versa for a male target.

### (a) Method

#### (i) Participants and stimulus material

A total of 40 participants (20 female, mean age _total sample_ = 21.8) from Middle East Technical University (Turkey) participated in the experiment as paid volunteers.

#### (ii) Procedure

In the instructions, the participants were presented with a story about two hypothetical friends: Selin (an unambiguously female name in Turkish) and Oktay (an unambiguously male name in Turkish) had similar taste. Selin and Oktay went shopping together and bought some stuff. On the screen, the pair of pictures of the choice objects (e.g. a watch, pair of glasses, iPhone, iPod, MacBook, Nespresso Machine, Scooter and Bag) was presented to the participants, where the objects were the same, but the colours were different. Overall, 16 pairs of objects were presented in 16 trials. Eight pairs included an object in black and the same object in white, whereas the remaining eight pairs included an object with a dark colour and the same object with a light colour. The participants’ task was to report which object was bought by the female character or by the male character. The participants were also informed that when they chose an object for Selin, they must take into account that the other object was bought by Oktay, and vice versa.

The participants’ task was manipulated between-participants: 20 participants reported the objects bought by the female character; the remaining 20 participants reported the objects bought by the male character. The location of the objects in the pictures was counterbalanced. The trials were also randomized in terms of their presentation order.

The experiment was conducted in single sessions. There were no time limits during the trials. Participants were asked to click on the picture to report their answers. Participants' eye movements were recorded by an SR Research EyeLink 1000 tower-mounted eye tracker with a sampling rate of 1000 Hz and reported average accuracy range of 0.25°–0.5°. The experiment took 10–15 min, including the eye tracker calibration. At the end of the experiment, participants were debriefed and thanked.

### (b) Results

#### (i) Analysis of choices

Since the response options in this experiment are also mutually dependent, we followed the same rationale used for the disambiguation data in the previous experiment. Namely, we calculated the average proportion of times each participant chose the black object, with 50% indicating an equal number of black and white objects chosen, 100% indicating only black objects chosen, and 0% indicating no black objects chose (i.e. 100% of white objects chosen). Nevertheless, we present the number of black and white objects chosen as a function of the task in table 2.

**Table 2.** Number of objects chosen as a function of task and colour.

	object colour	
task	black	white
choose for male	128	32
choose for female	28	132

A 2 (task: choose for female versus choose for male) × 2 (participants’ gender: males versus females) repeated measures ANOVA on the proportion of black objects chosen showed a large main effect of task, *F*_1,36_ = 140.19, *p* < 0.001, 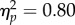. As hypothesized, participants chose more black objects when they were instructed to choose for the male (*M* = 0.80, s.e. = 0.04) than for the female character (*M* = 0.17, s.e. = 0.04). There was no interaction with participants' gender (*p* > 0.244), which suggests that this pattern is the same for male and female participants.

#### (ii) Gaze duration

A 2 (task: choose for female versus choose for male) × 2 (participants' gender: male versus female) × 2 (object colour: black versus white) mixed-model ANOVA revealed a strong interaction between task and object colour, *F*_1,36_ = 39.69, *p* < 0.001, 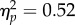. Planned comparisons showed that, as predicted, when instructed to choose for the male character participants looked longer at the black objects (*M* = 1286 ms, s.e. = 92 ms) than at the white objects (*M* = 1093 ms, s.e. = 94 ms), *t*_36_ = 3.98, *p* < 0.001, *d_z_* = 0.89. Conversely, when instructed to choose for the female character, participants spent more time looking at the white objects (*M* = 1053 ms, s.e. = 94 ms) than the black objects (*M* = 815 ms, s.e. = 92 ms), *t*_36_ = 4.93, *p* < 0.001, *d_z_* = 1.10. Additionally, there was a significant interaction between participants’ gender and object colour, *F*_1,36_ = 5.77, *p* = 0.022, 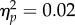. Namely, female participants looked longer at the white (*M* = 1117 ms, s.e. = 94 ms) than at the black objects (*M* = 1012 ms, s.e. = 92 ms), *t*_36_ = 2.17, *p* = 0.036, *d_z_* = 0.48. On the other hand, male participants looked longer at the black (*M* = 1088 ms, s.e. = 92 ms) than at the white objects (*M* = 1023 ms, s.e. = 94 ms); however, this difference did not reach statistical significance, *t*_36_ = 1.22, *p* = 0.230, *d_z_* = 0.27. The three-way interaction was not significant, which suggests that the pattern just described is similar for male and female participants. There were other theoretically uninteresting results, which we do not report here for space reasons.

#### (iii) Fixation count

A 2 (task: choose for female versus choose for male) × 2 (participants' gender: males versus females) × 2 (object colour: black versus white) mixed-model ANOVA showed an interaction between task and object colour, *F*_(1,36)_ = 69.48, *p* < 0.001, 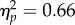. Planned comparisons showed that, when instructed to choose for the male character, participants made more fixations on the black objects (*M* = 4.92, s.e. = 0.29) than the white objects (*M* = 4.24, s.e. = 0.32), *t*_36_ = 5.05, *p* < 0.001, *d_z_* = 1.13. When instructed to choose for the female character, participants made more fixations on the white objects (*M* = 3.92, s.e. = 0.32) than the black objects (*M* = 3.01, s.e. = 0.29), *t*_36_ = 6.73, *p* < 0.001, *d_z_* = 1.50. The interaction between participants’ gender and object colour was again significant, *F*_1,36_ = 10.72, *p* = 0.012, 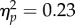. Again, female participants made more fixations on white objects overall (*M* = 4.34, s.e. = 0.32) than on the black objects (*M* = 3.91, s.e. = 0.29), *t*_36_ = 3.15, *p* = 0.003, *d_z_* = 0.70. Male participants did the opposite, namely, made more fixations on the black (*M* = 4.02, s.e. = 0.29) than the white objects (*M* = 3.82, s.e. = 0.32). However, this difference did not reach statistical significance, *t*_36_ = 1.47, *p* = 0.149, *d_z_* = 0.33. There were other theoretically uninteresting results, which we do not report here.

## 8. Discussion

The three experiments reveal that one feature guiding the categorization of females and males is the dimension of brightness. Universally, females have a lighter skin colour compared with males [5–8]. The three experiments reported here show a systematic relationship between the brightness dimension and the gender categories. As Experiment 1 shows, participants are faster in classifying female names in white as female names than when in black. The reverse pattern holds for male names. Male names in black are processed faster than male names in white. What is more, as Experiment 2 reveals, when presented rapidly with an ambiguous stimulus that is a white blob, then it is more likely that participants will classify it as a female name even though all they perceive is a blob in white. If, however, the blob is black, then it is classified as a male name. Finally, when participants are choosing light–dark or black–white objects for a female or a male then their gaze duration and the number of their fixations are more pronounced on the gender congruent objects. Thus, white- or light-coloured objects are attended to longer when the choice is for a female target, whereas attention is more pronounced for dark or black objects when the choice is for a male target. These three studies underline the generality of our findings, extending our earlier findings based on Dutch samples [1] to Portuguese (Experiments 1 and 2) and Turkish (Experiment 3) samples. Moreover, the current findings extend the generality of the proposed interface between the brightness dimension and gender by using a projective paradigm, namely the *disambiguation bias task* (Experiment 2) revealing that seeing merely a blob that is white or black is sufficient to activate a specific inference: white is female and black male!

A possible speculation that one can advance suggests that the sexual dimorphism of skin colour has become part of an implicitly acquired and used feature that marks males and females. It is possible that skin colour also exercises a force in shaping assumed male and female preferences for diverse objects. The brightness dimension captures attention differentially in relation to whether objects are more female (brighter objects)-specific or male-(darker objects)-specific. Thus, there seems to be an automatic tendency that may have been transduced from the sexual dimorphism of skin colour and anchored in a linguistic community. Obviously, this difference is noted across cultures in the different ways in which it has been represented across different communities in the world.

## 9. General discussion and implications

One might well ask how this differential processing is likely to come about. One possible avenue is via the critical adaptive mechanism that humans have, namely their ability to extract regularities from their complex and noisy physical and social environments. This ability to extract regularities is automatic and is referred to as ‘implicit learning’; as has been demonstrated by Reber [42,43] in his studies on the acquisition of synthetic grammars or ‘artificial grammar learning’, it takes place without intent or conscious awareness. These studies and later ones show that a brief and passive exposure to strings that are generated by an artificial grammar can lead to the acquisition of ‘implicit’ knowledge of the predictive relations within the stimuli typical of natural language characteristics [44,45]. The discovery of regularities in environmental input can also take place through statistical learning, the more complex forms of which can be akin to natural grammar learning. Thus, the patterns that are extracted can be rather simple (frequency count), or more complex (conditional probabilities). Indeed, language acquisition literature shows how infants display remarkable pattern-learning qualities [44,46].

Such extracted regularities serve as anticipatory competencies and have important adaptive functions in that they prepare us to meaningfully relate and act upon the ever-changing dynamic features of our world to the extent that we can make them predictable (regulatory anticipation). We are able to represent these extracted regularities by transducing them into patterns of neural activity and associated action tendencies. This does not mean that we have conscious access to how we engage in ‘correct’ actions and, moreover, even when we do have an ‘account’ of the nature of the regularity driving a particular adaptive action, such accounts constitute representations on a different medium.

The implicit knowledge acquired via regularity extraction processes are transduced into a notional system, as for instance, in the case of metaphoric representations of gender. Culture adds layers of interpretation upon white and black in connection with gender. Thus, white is the colour most commonly associated with innocence and the virgin is the epitome of purity, grace and chastity. The association between white, honesty and cleanliness has its Western origins in Biblical times when the sacrifice of white animals, like lambs, was a manner of making amends for one's sins. The white unicorn, which only a virgin could capture, was a prominent symbol of innocence, purity and grace in medieval times. In current Western culture, the bride wears a white wedding gown, a so-called white wedding, or one of a light colour. Indeed, it appears that this tradition was consolidated with the marriage of Queen Victoria to Albert of Saxe-Coburg after 1840. Godey's Lady's Book was one of the most influential magazines at around the time of this momentous wedding, reaching a circulation of approximately 150 000 around 1860. Referring to European brides, it pronounced in 1839 that: ‘Custom has decided, from the earliest ages, that white is the most fitting hue, whatever may be the material. It is an emblem of the purity and innocence of girlhood, and the unsullied heart she now yields to the chosen one’. The association between white and purity is also to be found in ancient civilizations. For instance, in Greek civilization white was associated with mother's milk. Milk was regarded as a sacred substance in the Talmud and in ancient Egypt white was connected with the goddess Isis. In India, white symbolizes purity, divinity, detachment and serenity. In Anatolia, white symbolizes nobility and supremacy. Across different eras and cultures, the colour white has been associated with purity, cleanliness and femininity.

Black, by contrast, is culturally often seen as the colour of authority and seriousness and notably all the symbols of black that have to do with authority and seriousness are associated with the male gender. Black represents power, law and authority as in the case of academic robes for graduates, in general, and professors of ceremonial occasions in some cultures such as The Netherlands or Germany. Similarly, the seriousness and authority associated with black are also symbolized in black tuxedos that are often worn on formal occasions such as black tie functions. In many countries in Europe, professors wear long black gowns with wide sleeves, a tradition that can be traced back to the everyday clothes of the scholars in the Middle Ages. Black in this context symbolizes knowledge and authority as well as power and law, as can be seen in many countries where judges and magistrates wear black robes. Indeed, police uniforms in many countries were black until the twentieth century. Government officials' cars or big company limousines are black as displays of authority and power. Indeed, business suits and costumes that judges or priests wear are predominantly black. Also, Christian priests and performers of classical music are all adorned with black attire representing a sombre, serious and authoritative stance. Yet, white is not a symbol for the absence of authority and seriousness.

Such transduced representations maintain an inherent duality, namely, a regularity that is inherent to the nature of the environment that we perceive and from which implicit knowledge is derived. The second type is inherent to the transduced explicit representation that retains all or some features of the implicit knowledge. Regularities such as the sexual dimorphism of skin colour constitute perceptual regularities of our environment and are implicitly acquired. However, they often become layered with cultural artefacts that emerge as a consequence of the *culturally* adaptive significance of observed and implicitly registered regularities.

## Endnote

1 We analysed the proportion of correct responses to names and nouns separately. Regarding the names, we found a significant interaction between names and colour, *F*_1,35_ = 4.16, *p* = 0.049, . Planned comparisons showed that, for female names, participants were more accurate when these where presented in white (*M* = 0.86, s.e. = 0.05) than in black (*M* = 0.83, s.e. = 0.06), *t*_35_ = 3.30, *p* = 0.003, *d*_z_ = 0.55. For male names, there was no difference between names presented in black (*M* = 0.86, s.e. = 0.05) and in white (*M* = 0.86, s.e. = 0.05), *t* < 1. As for the nouns, we again found a significant interaction between names and colour, *F*_1,35_ = 5.21, *p* = 0.029, ; however, none of the planned comparisons reached statistical significance (*p* > 0.050).

## Data accessibility

This article has no additional data.
